# Inhibition of *Plasmodium berghei* Development in Mosquitoes by Effector Proteins Secreted from *Asaia* sp. Bacteria Using a Novel Native Secretion Signal

**DOI:** 10.1371/journal.pone.0143541

**Published:** 2015-12-04

**Authors:** Nicholas J. Bongio, David J. Lampe

**Affiliations:** Department of Biological Sciences, Duquesne University, Pittsburgh, Pennsylvania, United States of America; University of Camerino, ITALY

## Abstract

Novel interventions are needed to prevent the transmission of the *Plasmodium* parasites that cause malaria. One possible method is to supply mosquitoes with antiplasmodial effector proteins from bacteria by paratransgenesis. Mosquitoes have a diverse complement of midgut microbiota including the Gram-negative bacteria *Asaia bogorensis*. This study presents the first use of *Asaia* sp. bacteria for paratransgenesis against *P*. *berghei*. We identified putative secreted proteins from *A*. *bogorensis* by a genetic screen using alkaline phosphatase gene fusions. Two were secreted efficiently: a siderophore receptor protein and a YVTN beta-propeller repeat protein. The siderophore receptor gene was fused with antiplasmodial effector genes including the scorpine antimicrobial peptide and an anti-Pbs21 scFv-Shiva1 immunotoxin. *Asaia* SF2.1 secreting these fusion proteins were fed to mosquitoes and challenged with *Plasmodium berghei*-infected blood. With each of these effector constructs, significant inhibition of parasite development was observed. These results provide a novel and promising intervention against malaria transmission.

## Introduction

In 2013, there were approximately 198 million new cases of human malaria, and the disease was estimated to have caused 584,000 deaths worldwide, the majority among children under 5 years old in sub-Saharan Africa [1]. Currently, there are 106 countries and territories where malaria is transmitted meaning that 3.3 billion people are at risk of becoming infected with malaria every year [1].

Given its widespread nature, a great deal of research focuses on malaria prevention and treatment. The two most common methods of control are antimalarial drugs that target the *Plasmodium* spp. parasites in the human host and the use of insecticides to manage mosquito vector populations [2, 3]. Despite decades of research providing new drugs and interventions, malaria is still a large problem in many developing nations across the world, although its incidence is decreasing [1]. Malaria remains a challenging disease to control since *Plasmodium* spp. rapidly evolve resistance to new drugs, and the drugs themselves are expensive to produce and distribute in the quantities necessary to cure the millions of annual cases [4, 5]. Similarly, the *Anopheles* spp. mosquitoes that transmit the parasite have evolved resistance to the insecticides intended to target them [6].

The *Plasmodium* parasites that cause malaria have a complex life cycle requiring both human and mosquito hosts [7]. Fertilization within the mosquito host and subsequent invasion of the mosquito midgut present the greatest bottlenecks to *Plasmodium* transmission [8]. In field-caught malaria vectors, there are typically between 0–5 oocysts per midgut despite the ingestion of thousands of parasites in a typical infective blood meal [9]. This dramatic bottleneck in the number of parasites in the mosquito host is due largely to the killing of the parasites by the mosquito’s immune system [10]. The parasite life stages within the mosquito have, therefore, been targeted for transmission-blocking experiments in attempts to stop the development of the parasite before the anopheline host becomes infective. These strategies aim to render the vectors incapable of transmitting *Plasmodium* rather than killing them.

In principle, there are two ways to reduce or eliminate the ability of mosquitoes to transmit *Plasmodium*. The first is to create transgenic mosquitoes that express some effector to block the development of *Plasmodium*. Many such effectors are known including SM1 (salivary gland midgut peptide 1) which blocks the invasion of the midgut epithelium by ookinetes and salivary glands by sporozoites [11]; scorpine (a peptide component of scorpion venom) which kills by lysing parasites [12]; PLA2 (honey bee venom phospholipase 2) which kills by preventing the invasion of the midgut by ookinetes [13]; and a single chain antibody (scFv) against *P*. *berghei* ookinete surface protein 21-Shiva 1 fusion protein which lyses ookinetes after binding to them [14]. Transgenic mosquitoes that express some of these effectors have demonstrated resistance to *Plasmodium* infection [15]. However, the generation of transgenic mosquitoes for malaria control is not trivial, and replacing the natural population of vector mosquitoes in the wild requires driving the effector gene into multiple populations to achieve high allele frequencies. Additionally, *Plasmodium* is not transmitted by a single mosquito species; at least 20 species transmit *Plasmodium* efficiently [16]. Even within a single vector species, there can be distinct sexually-isolated molecular types that do not interbreed, as is the case with the *Anopheles gambiae* molecular forms [17]. For these reasons, transgenic mosquitoes may be difficult to utilize in field applications.

A simpler approach to deliver antiplasmodial effector molecules to mosquitoes is to produce bacterial strains which are capable both of inhabiting the midgut of diverse mosquito species and of rapidly spreading among wild mosquito populations [18]. This strategy is termed paratransgenesis and is the modification of symbiotic microorganisms residing within a host in order to alter that host's phenotype, in this case the ability to transmit a disease-causing organism [19]. The first successful demonstration of this technique was the use of paratransgenic *Rhodococcus rhodnii* in the triatomine bug, *Rhodnius prolixus*, to combat Chagas disease [20]. Our previous work targeted malaria by engineering the bacterium *Pantoea agglomerans* to secrete antiplasmodial proteins into the midgut of the *Anopheles* host of *Plasmodium* using the Type I *E*. *coli* hemolysin secretion system [15]. Two additional studies also targeted malaria: an *Anopheles* densonucleovirus was modified to produce green fluorescent protein within the *Anopheles* host to demonstrate its potential for delivery of antiplasmodial effectors [21]; and the fungus *Metarhizium anisopliae*, a natural pathogen of mosquitoes, was modified to produce the antiplasmodial peptide SM1 and the antimicrobial scorpine and proved capable of reducing sporozoite counts by 98% [22]. Finally, some select bacterial strains isolated from mosquitoes are capable of blocking *Plasmodium* development without any further genetic manipulations [23].

Bacteria chosen for paratransgenesis in mosquitoes must be selected from among the group of symbiotic species that inhabit the mosquito midgut. Previous experiments used *P*. *agglomerans* to secrete antiplasmodial proteins into the midgut environment [15]. This species secreted antiplasmodial proteins in high amounts and demonstrated effective parasite killing in vivo. *P*. *agglomerans*, however, has no drive mechanism that will cause the paratransgenic organism to remain in the mosquito population long-term or to spread the bacteria within a population of mosquitoes from one individual to another. Bacteria of the genus *Asaia*, first discovered in plant nectar, are much better candidates for paratransgenesis [24, 25]. The bacterial strain *Asaia* sp. SF2.1, which was originally isolated from *Anopheles stephensi* mosquitoes, has been successfully cultured and manipulated in the lab [26]. These bacteria are prime candidates for paratransgenesis due to the ease with which they can be introduced into a population of mosquitoes and the rate at which they can spread throughout a population [27]. *Asaia* is associated with many tissues of the mosquito body; it can be found inhabiting the reproductive organs, the salivary glands, and the midgut [26]. All three of these organs are important for paratransgenic malaria intervention. *Asaia* sp. SF2.1 has also been demonstrated to be transmitted from female mosquitoes to their progeny by egg smearing, in which the mother paints bacteria on the surface of eggs, and the bacteria passed on survive until adulthood [26, 27]. These bacteria can also be passed from male to female during copulation since they inhabit the reproductive organs of the mosquito host [27].

*Asaia* can represent a large proportion of the midgut mosquito bacterial population, in some cases encompassing 60% of all bacteria as identified by 454 sequencing [28]. *Asaia* has been identified in many species of *Anopheles* mosquitoes, and it seems to be an integral part of the host's midgut microbiome necessary for the health of the mosquito host [29]. In addition, *Asaia* contribute to overall health during larval development by increasing the transcription of certain host genes that allow the larvae to develop more rapidly [29]. These aspects of *Asaia* microbial ecology make this bacterium an attractive candidate for paratransgenesis [26, 30].

In this study, we conducted a genetic screen to isolate native *Asaia* protein secretion signals in order to construct paratransgenic strains capable of delivering antiplasmodial effector proteins into the midgut of *A*. *stephensi* mosquitoes. Of the 16 unique signal sequences identified in the screen, one allowed the creation of *Asaia* strains that significantly reduced both the number of *Plasmodium berghei* oocysts in *A*. *stephensi* and the prevalence of mosquitoes carrying parasites. Together with other genetic tools under development for *Asaia*, this may allow the species to be developed as a practical paratransgenic platform to combat malaria.

## Methods

### Media and antibiotics

Top10 F' *E*. *coli* cells were used during the molecular cloning and plasmid propagation stages of the experiments. The Top10 F' cells were cultured in standard Luria Bertani (LB) broth (10 g Tryptone, 5 g NaCl, 5 g yeast extract, in a final volume of 1 L distilled water) or LB agar (LB broth plus 15 g/L agar). For selection of plasmids with kanamycin, media was supplemented with 30 μg/ml kanamycin. All *E*. *coli* cultures were grown at 37°C, with agitation for liquid broth cultures.

*Asaia* SF2.1 bacteria were cultured in mannitol broth (5 g yeast extract, 3 g peptone, 25 g mannitol, in a final volume of 1 L distilled water) or mannitol agar (mannitol broth plus 15 g/L agar). Media was adjusted to pH 6.5 before sterilization. For selection of plasmids with kanamycin, media was supplemented with 120 μg/ml kanamycin. All *Asaia* cultures were grown at 30°C, with agitation for liquid broth cultures.

All bacterial strains and plasmids used in this study are described in Table 1.

**Table 1:** Strains and Plasmids used in this study.

**Strains**	**Relevant Characteristics**	**Source**
*E*. *coli* Top10 F'	F´{lac*Iq*, Tn*10*(TetR)} *mcrA* Δ(*mrr*-*hsdRMS*-*mcrBC*) Φ80*lacZΔM15* Δ*lacX74 recA1 araD139* Δ(*ara leu*) 7697 *galU galK rpsL* (StrR) *endA1 nupG* (from life technologies #C3030-03)	[31]
*E*. *coli K-12 MG1655*	F- lambda- *ilvG*- *rfb*-50 *rph*-1	[32]
*Asaia bogorensis*	Isolate from orchid tree flower, Indonesia (ATCC BAA-21)	[24]
*Asaia* sp. *SF2*.*1*	Wild type strain isolated from *Anopheles* mosquitoes.	[26]
**Plasmids**	**Relevant Characteristics**	**Source**
pBBR1MCS-2	Kan^R^, pBBR origin, *oriT*, MCS, used for plasmid construction	[33]
pNB20	pBBR1MCS-2 with the *lacZα* gene replaced by a new MCS.	This study
pNB51	pNB20 w/ promoter region changed to P_nptII_	This study
pNB90	pNB51 w/ '*phoA* insert used for genomic library construction	This study
pNB91	Random genomic library of *Asaia* DNA-‘*phoA* fusions	This study
pNB92	pNB51 w/ '*phoA* insert and MCS for gene fusion construction	This study
pNB93	pNB92 w/ YVTN beta-propeller repeat gene cloned	This study
pNB95	pNB92 w/ siderophore receptor gene cloned.	This study
pNB96	Siderophore receptor—Pro-EPIP–‘*phoA* effector construct	This study
pNB97	Siderophore receptor—scorpine–‘*phoA* effector construct	This study
pNB99	Siderophore receptor—PLA2 –‘*phoA* effector construct	This study
pNB101	Siderophore receptor—prochitinase–‘*phoA* effector construct	This study
pNB102	Siderophore receptor—anti-Pbs21 scFv-Shiva1 –‘*phoA* construct	This study
pNB103	Siderophore receptor—Shiva1 –‘*phoA* effector construct	This study

### Expression plasmid construction

The expression vector used in multiple host bacteria was constructed using the backbone of pBBR1MCS-2, a broad-host range plasmid [33]. The sequences of all primers used in this study are listed in S1 Table.

The *lacZα* gene of pBBR1MCS-2 was replaced by bacterial recombination with a new cloning site. Primers “pBBR rec F” and “pBBR rec R” were designed to amplify the ampicillin resistance gene (*bla*) encoding beta-lactamase and extend this to have a translational enhancer preceding the ORF, an NdeI restriction site at the beginning of the gene, and five other unique restriction sites following the gene. The primers also included 40 nucleotides of homology to the pBBR1MCS-2 plasmid to mediate recombination. The *bla* gene was amplified by PCR from pSC189 [34] using these primers and purified by gel-extraction using a Zymo kit (Zymo Research, Irvine, CA #D4002). The pBBR1MCS-2 plasmid was purified from *E*. *coli* using a Qiagen miniprep kit (Qiagen, Germantown, MD, #27104). The pBBR1MCS-2 plasmid was linearized by digestion with the EcoRI and BamHI restriction enzymes to completion.

Fifty ng of the linear plasmid and 50 ng of the PCR product were co-transformed into MG1655 *E*. *coli* cells, which were incubated in non-selective LB media for 1.5 h at 37°C to allow for the bacteria to recombine the two DNA fragments. This culture was plated on selective LB agar medium containing ampicillin. The resulting colonies from this selection contained the new plasmid named pNB20, which now had a specific set of restriction sites with which to modify the expression cassette.

The pNB20 plasmid was further modified by changing the promoter region for beta-lactamase to the neomycin phosphotransferase promoter (P_nptII_). This promoter is constitutively active and has high activity in *Asaia* sp. as demonstrated by its effective use expressing green fluorescent protein [26]. The P_nptII_ region of pHM2-GFP [26] was amplified by standard PCR using primers that add NsiI and NdeI restriction sites to the ends of the amplicon (primers “PnptII F NsiI” and “PnptII R NdeI”). This PCR product and pNB20 were each digested to completion with NsiI and NdeI restriction enzymes, and the plasmid was treated with calf intestinal phosphatase. The PCR product was ligated into the linearized plasmid using T4 DNA ligase at 16°C overnight. The ligated DNA was transformed into Top10 F' *E*. *coli* cells and selected on LB media containing ampicillin and kanamycin. Clones isolated from this selection were verified by sequencing to ensure there were no mutations introduced. The new plasmid created was named pNB51. The sequences of the plasmids created in this study were deposited in Genbank under accession numbers KT826597-KT826603.

### Selection of secreting strains of bacteria

To identify the secretion of protein constructs in bacterial colonies, media was supplemented with 5-bromo-4-chloro-3'-indolyl phosphate (BCIP). *E*. *coli* was screened on LB media supplemented with 25 μg/ml BCIP. *Asaia* was screened on mannitol agar supplemented with 25 μg/ml BCIP and 25 μg/ml Na_2_HPO_4_. Sodium phosphate was included in the media as an inhibitor of the natural phosphatase activity contained in wild type *Asaia* to reduce false positives.

### Genomic library construction and screening

The *E*. *coli* ‘*phoA* gene was inserted in pNB51 in such a way that genomic DNA could be cloned 5' to it to allow gene fusions. ***‘*** *phoA* was amplified by PCR from *E*.*coli* K12 genomic DNA using primers “PhoA F NdeI” and “PhoA R PacI.” These primers amplify *phoA* from amino acids 26 to 471, which excludes the native PhoA secretion signal. The product was directionally cloned into the expression vector pNB51 replacing the beta-lactamase gene, to create a plasmid with a cloning site usable for gene fusions with ‘*phoA*, named pNB90.

**Fig 1:**
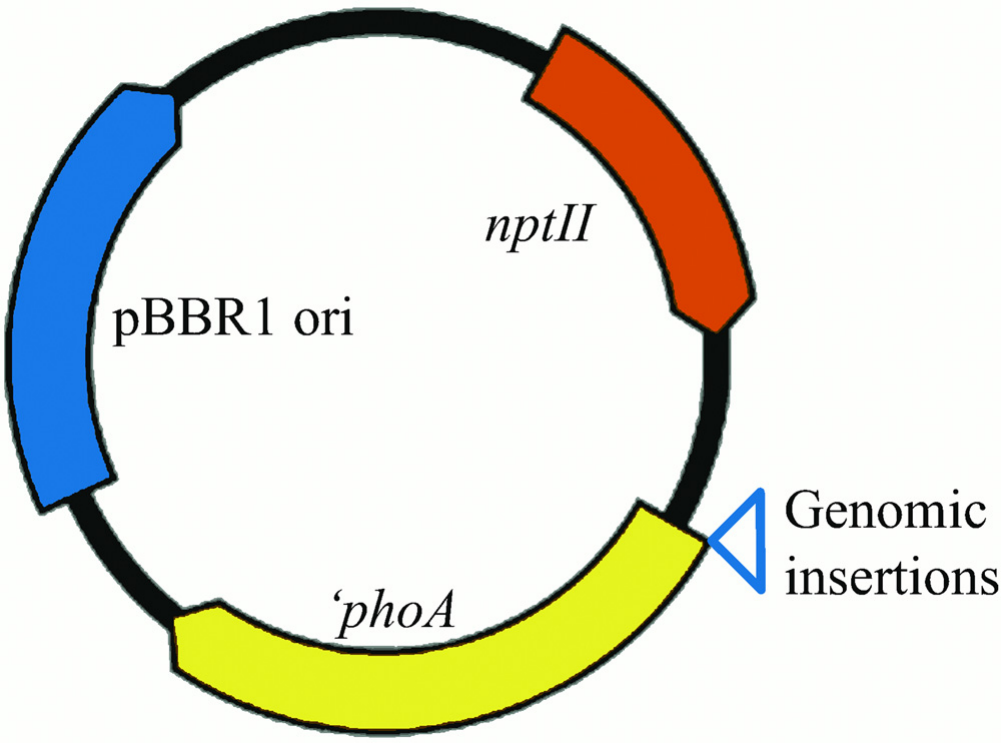
pNB90 backbone for the genomic library screen. Random size-selected genomic fragments were cloned 5’ to ‘*phoA* to create a library that could be screened to detect sequences capable of mediating secretion of PhoA. pBBR1 *ori* = origin of replication; *nptII* = neomycin phosphotransferase II conferring kanamycin resistance; *‘phoA* = *E*. *coli* alkaline phosphatase gene with no native signal sequence.

*Asaia* genomic DNA was partially digested with the MseI restriction enzyme and the expression vector pNB90 was fully digested with the NdeI restriction enzyme. Digested genomic DNA was size-selected for fragments 2–4 kb in size by gel electrophoresis, recovered, and ligated into the NdeI site of pNB90 to create a fusion library between ‘*phoA* and *Asaia* genomic inserts (Fig 1). The library was transformed into *E*. *coli* Top10 F' electrocompetent cells and plated on LB agar containing kanamycin and BCIP, and grown 12 h at 37°C.

The library was screened in *E*. *coli* due to the inefficiency of DNA transformation into *Asaia* at a rate suitable for library screening. Approximately 0.9% of colonies turned blue after 1 day of growth at 37°C. More than 200,000 clones were collected and pooled to provide over 100-fold coverage of the 3.53 Mb genome, and these were stored in 50% glycerol at -80°C to make the library.

Individual blue colonies were inoculated into 5 ml LB broth supplemented with kanamycin and grown at 37°C for 12 h. Plasmid DNA was extracted from these cultures using a Qiagen plasmid miniprep kit. One hundred and sixty blue clones were plasmid prepped and sequenced using the primer “PhoA seq R1”. Each sequence read was identified by a BLAST search (NCBI) for the closest related homologous protein identity.

One plasmid for each unique clone was transformed into *Asaia* cells. These were plated on selective media to confirm that secretion functions similarly to the activity seen in *E*. *coli*.

### ELISA to detect PhoA in *Asaia* cell culture fractions

*Asaia* expressing the PhoA secretion constructs were grown to OD_600_ of 1.5 and then centrifuged. Supernatant, live cell, and cell lysate fractions were isolated and bound to wells in a 96-well plate overnight at 4°C. These plates were washed three times with Tris-buffered saline (TBS) (50mM Tris-CL, 150 mM NaCl, pH 7.5) then blocked by adding 200 μl of 2% BSA TBS (TBS with 2% w/v fraction V BSA) and incubating 2 h at room temperature. This was washed again three times with TBS, then 100 μl of a 1/3000 dilution of anti-BAP-HRP (bacterial alkaline phosphatase) antibody (Abcam, Cambridge, England, ab7319-1) in 2% BSA TBS was added to each well and incubated for 1 h at room temperature. The plate was washed 8 times with TBS-Tween20 0.1% for 2 min per wash. To visualize the protein, 50 μl of 1-Step Ultra TMB-ELISA (Thermo Scientific, Ipswich, MA, #34028) was added and incubated for 10–20 min at room temperature. The reactions were stopped by the addition of 50 μl of 2M H_2_SO_4_, and the absorption at 450 nm was measured using a plate reader.

### PhoA activity assay

*Asaia* expressing the PhoA secretion constructs were grown to OD_600_ of 1.5, and then centrifuged. Supernatant, live cell, and cell lysate fractions were isolated and bound to wells in a 96-well plate overnight at 4°C. PhoA activity was detected by rinsing the plates with PBS, and adding 100 μl of p-nitrophenyl phosphate (PNPP) substrate (Sigma Aldrich, St. Louis, MO, #P7998). PhoA cleaves the phosphate group from PNPP substrate, producing a yellow color. Absorption at 400 nm was measured using a plate reader.

### Western blot detection of secreted proteins from *phoA* library clones

*Asaia* liquid cultures were grown to an OD_600_ of 1.5. Protein samples were prepared from these by centrifuging 1 ml of the culture at 12,000 RPM in a desktop centrifuge. From the supernatant fraction, 750 μl was removed, mixed with 250 μl of 3X Laemmli sample buffer (BioRad, Hercules, CA, #161–0737), and boiled for 10 minutes. Eight μl of Colormark Plus Prestained Protein Ladder (New England Biolabs, Ipswich, MA, #p7712), and 20 μl of each protein sample were loaded into wells of a 5%-stacking and 10%-separating polyacrylamide gel. Proteins were separated by electrophoresis at 120 V for 1 h, then transferred onto a PVDF membrane in a BioRad transfer apparatus using Tris-Glycine transfer buffer (25mM Tris, 150mM glycine, 10% methanol) at 95 V for 75 minutes. The membrane was blocked with a 4% bovine serum albumin (BSA) TBS-T solution (50 mM Tris-CL, 150 mM NaCl, 0.1% Tween20, 4% w/v fraction V BSA, pH 7.5) for 3 h at 4°C with agitation. The blocking buffer was replaced with the primary antibody solution containing rabbit polyclonal anti-bacterial alkaline phosphatase 1:50,000 (Abcam, Cambridge, England, ab7319-1) in blocking buffer and incubated overnight at 4°C. The next day, the membrane was washed four times in TBS-T for 15 min each time. Following washes, the secondary antibody was applied, goat anti-rabbit HRP (BioRad, Hercules, CA, 170–5046), and the blot was incubated for 1 h at room temperature. The wash steps were repeated, then 2 ml of Supersignal West Femto Maximum Sensitivity Substrate (Thermo Scientific, Waltham, MA, # 34095) was added to the blot and incubated for 5 minutes. The blot was exposed to X-ray film (Thermo Scientific, Ipswich, MA, #34090) and developed using an automatic developer.

### Cloning of antiplasmodial effector genes into secretion constructs

A new plasmid was generated to modify the identified secretion constructs to include a cloning site into which antiplasmodial effector genes could be inserted. First, the ‘*phoA* gene was amplified by PCR using primers “PhoA F NdePacSbf” and “PhoA R Fse”. This PCR product and the pNB51 plasmid were digested to completion with NdeI and FseI restriction enzymes, and the plasmid was treated with calf intestinal phosphatase. The PCR product was ligated into the linearized plasmid using T4 DNA ligase at 16°C overnight. The ligated DNA was transformed into Top10 *E*. *coli* cells and selected on LB media containing kanamycin. Clones isolated from this selection were verified by sequencing to ensure there were no mutations introduced. The new plasmid created was named pNB92. This plasmid is identical to pNB90, but with the addition of the PacI and SbfI restriction sites for in-frame insertion of effector genes into the fusion protein construct.

The siderophore receptor gene was amplified by standard PCR using primers “siderophore F NdeI” and “siderophore R MYC PacI,” and the YVTN beta-propeller gene was amplified using primers “YVTN F NdeI” and “YVTN R MYC PacI.” The PCR products were directionally cloned into pNB92 by restriction enzyme digestion and ligation. The resultant gene constructs contain the siderophore receptor or YVTN beta-propeller gene fragment, a MYC epitope tag, a cloning site for effector molecules, and ‘*phoA*. The YVTN and siderophore plasmids were named pNB93 and pNB95, respectively. Insertions were sequenced to verify that there were no mutations.

Antiplasmodial effector genes were cloned in-frame with the signal sequences, including prochitinase peptide, PLA2, enolase-plasminogen interacting protein (EPIP)4, anti-Pbs21 scFv-Shiva1, prochitinase-EPIP, and scorpine (see reference 15, S1 Table for a description of each). Each gene was amplified by PCR using primers that add PacI and SbfI restriction digest sites to either end of the amplicon. These PCR products were directionally cloned into pNB93 and pNB95 by restriction enzyme digestion and ligation as described previously to create a set of antiplasmodial secretion constructs. Clones were selected on LB agar containing kanamycin. The YVTN beta-propeller protein constructs would not allow *Asaia* to grow properly when transformed, possibly due to lethality of the cloned constructs, therefore following the molecular cloning and sequence verification, *Asaia* SF2.1 cells were transformed with each of the siderophore receptor effector plasmids only, and they were selected for on mannitol agar supplemented with kanamycin.

### Parasite inhibition testing in *A*. *stephensi* mosquitoes

*A*. *stephensi* (Dutch strain) were maintained on 5% (w/vol) sucrose solution at 29°C and 70% humidity with a 12h day:12h night light cycle. *P*. *berghei* strain ANKA2.34 was maintained by passage through 7–8 week-old female Swiss Webster mice using standard procedures [35]. This study was carried out in strict accordance with the recommendations in the Guide for the Care and Use of Laboratory Animals of the National Institutes of Health. Experiments involving mice were approved by the Institutional Animal Care and Use Committee of Duquesne University (Protocol # 1207–07). All surgery was performed using anesthesia as outlined below, and all efforts were made to minimize suffering.

Two-day old *A*. *stephensi* adults were collected and separated into cups with screen lids containing 50 female mosquitoes each. *Asaia* expressing the siderophore-effector protein secretion constructs and a control strain secreting PhoA alone were fed to individual mosquito cups for 48 h by cotton balls soaked with a mixture containing 1x10^9^ cells/ml bacteria in a 5% sugar solution.

The parasitemia of a *P*. *berghei* infected female Swiss Webster mouse was determined by blood smear. A drop of blood was taken from the mouse's tail and placed on a slide. This was smeared with the edge of another glass slide to create a thin layer of blood. This slide was fixed in methanol for ten seconds, then transferred to a 10% Giemsa stain solution: 5 ml Giemsa stock solution (Ricca Chemical Company, Arlington, TX, #3250–4) plus 45 ml phosphate buffer (0.35g KH_2_PO_4_ plus 0.5g Na_2_HPO_4_ in a final volume of 500 ml distilled water). This was allowed to stain for 30 min, after which it was rinsed with tap water and dried. Parasitemia was determined by counting the number of infected cells and dividing by the total number of cells in a given microscope field. Full fields were counted until at least 500 cells were evaluated.

After determining the parasitemia, the mouse was sacrificed using a CO_2_ chamber, and the total blood volume was removed by cardiac puncture surgery with a heparinized needle (100 μl of 1 mg/ml heparin in PBS, 26 G needle). This blood was diluted with phosphate buffered saline to reach 5x10^7^ infected cells per 200 μl. A volume of 200 μl of the diluted blood was immediately injected into three naïve mice intraperitoneally.

The newly infected mice were checked for infectivity by exflagellation testing 2–3 days post-injection. A drop of blood was extracted from the mouse's tail, and 2 μl of the blood was combined with 2 μl heparin (1 mg/ml heparin in PBS) and 6 μl exflagellation media (1 pkg of RPMI 1640 medium (1 L / pkg Gibco, Thermo Scientific, Waltham, MA, #23400–021) with 2 mM Hepes, 2 mM glutamine in 1 L distilled water pH 8.4, supplemented with 2 g sodium bicarbonate, 50 mg hypozanthine, and 100 μM xanthurenic acid). The mixture was incubated at 21°C for 10 min. After incubation, the entire 10 μl was transferred to a microscope slide and covered with a coverslip. At 100X magnification, exflagellation events were counted per field, in uncrowded fields that show only a single layer of cells; these are estimated at 500 cells per field.

The mosquitoes that were previously fed the paratransgenic *Asaia* strains were given a *P*. *berghei* infective blood meal from a mouse with exflagellation rates of between 2–3 events per field. The mice were anesthetized by intraperitoneal injection of 150 μl of anesthetizing agent (Ketaject [100 mg/ml] 2 ml, acepromazine [10 mg/ml] 1 ml, saline [0.9% w/v NaCl] 7 ml). The mouse was placed on the screen top of each cup consecutively, and mosquitoes were allowed to feed for 6 min. Multiple trials were performed, each with a control cup fed *Asaia* expressing a plasmid with no effector, and two cups that were fed different antiplasmodial *Asaia* strains. The infected mosquitoes were kept at 21°C with 10% sugar solution for two weeks. Midguts were dissected and stained with 0.2% mercurochrome which stains the oocysts. The number of oocysts per midgut was counted using a light microscope.

## Results

### Genetic screen for secreted proteins in *Asaia*

A genetic screen for secreted *Asaia* proteins identified sixteen unique genes encoding proteins that were predicted to be either secreted from the cell or membrane-bound (Table 2). Less than 1% of the clones in the genomic library produced a blue phenotype. This is a reasonable number of clones to obtain considering that half of the inserted DNA will be in the incorrect orientation, and two-thirds of the remaining inserts will be in the incorrect reading frame for translation.

**Table 2:** Proteins identified from a genetic screen for secreted *Asaia* proteins.

Protein identification	Amino acid sequence	Predicted secretion pathway
Amino acid ABC transport permease (Aap)	MALPXSRAMSRFHDSVDXVLGDMMSDGTLQTILRRWNLWTPEMAAMTGDPQRCVRCLLFAWLRYRDAMRSTARLGGAVPPLSRISAHHRQGGGADPCGVGPFHDP	No signal identified.
Lysine 2,3-aminomutase (Amu)	MLSPRRLRHIIEALSAMPHIQTIRIHSRVPVADPARITSAMLDALETDRALWIVLHANHASEMTGQARAAIRQIQSRAIPVLSQSVLLRGVNDTEEALEALLRAFVTARIKPYYLHQLDPAPGTSHFHVPI	No signal identified.
Carbohydrate porin (Chp)	MDQMIWRSHTDPNRTISLFGRAMGAPQSDRVPIDFSLNFGLTFNDPLPYRTDDTFGIGMGYTHVSGALANYDRAVRRYSGAYSPTQGGETYVELTYQYQFTGWMQWQPDFQYIFNPGGGIPNPSHPDRRL	No signal identified. Transmembrane domain predicted.
Cellulose synthase subunit AB) (Csy)	MKHVRHSIAFLESWIDDAHHSPARTAIKTGLISFAILCMVIAAFVHL	No signal identified. Transmembrane domain predicted.
Cell wall-associated hydrolase (Cwh)	MLVRSLVSRPYGWGNYNFYNDCSAELRSLLIPFGILMPRNSLAQIQATSRTVDLGKEDVEARLDYLV	No signal identified.
Hypothetical protein (*Gluconacetobacter* fimbrial assembly family protein) (Faf)	MQRDVTLPLAAEQDLAAILAYEMDRLTPFDAEALFWDFIVLRRDEALGQIMLRLSVVPQAPLRPLFERLHLLEAHPQAIADETGETLIRQPVARPLVTRLSDPRLALPLGGCTLLACLLLGLFWHQSRVLSGYERQIDALRGPALE	No signal identified.
Glucose dehydrogenase (membrane-bound PQQ-dependent dehydrogenase)(Gdh)	**MTMLAVRADFTACRNRQVVSFSGA**VTDNNSTKEPSGVTRAFDLFTGKLVWVFDPSNPDPNEMPSGDHKYVANSPNSWITASYDANLGLVYIPTGVQTPDQWGGNRTPDAERYASSVLALHADTGKLAWSYQTVHHDLWDMD	Sec-dependent (0.718 reliability score)
Multidrug ABC transporter (Mdt)	MEMSIIRSIDVELGQYVKKGQVLAHLDPTITKADIVNLKAQRDSYQATINRLHAEAEGKTFTPDL	No signal identified.
Major facilitator transporter (Mft)	MMLARLAHLTYNPHSSFCKINSFSFKLRIYRKMSQLTSHDNRLVGPYGYSALAIAALIFFAMGFVTWL	No signal identified. Transmembrane domain predicted.
Membrane protein glycerol transporter (Mgt)	**MTQITVVLLFGITTIH**GVTQVGDFYA	Sec-dependent (0.983 reliability score)
Peptide ABC transporter permease (Ptp)	MIRLALRLRGLSGSGF	No signal identified.
Ribonuclease I (secreted ribonuclease T2 family) (Rns)	MGTYLAERAGLRVRHDDLMAFFRTASQTTLPRALQLRCETDHEGRIVLTQLWFTLAPGKMHLFPAAESYLTSPQNQDNCPAEFWV	No signal identified.
TonB-dependent receptor (Tdr)	**MHRYGNLLLSMGLNINRTTLTHNGLSATGTPLLNA**QTTAYLTSESPRSKIVLNAYYTLGNWDVNLRQSRYGQTVGMLTYQDWTPASAICPINGKALRGSNVCFAQFKNTPRWLTDLEIGYRFNQHWHVAVGANNIF	Sec-dependent (0.982 reliability score)
Hypothetical protein (*Xanthobacter* TonB-dependent hemoglobin receptor) (Thr)	**MPAYRAITLAAANLTKYNLTTLGFEA**DNTSQFPIGPVLASLNYGGEYYHDSVKTKDQTGYEGSTPSGGRGVGSAFTQLALNWKIVQLTGALRYDTYHASGSGV	Sec-dependent (0.949 reliability score)
Hypothetical protein (*Acetobacter* outer membrane siderophore receptor) (Tsr)	**MTLLPRETHDSGRHNPIVAPSTRRKRPLAAGLMSATACVALLHFVSDARA**QSVSASETSTIPVNAAKAPAKSKVKVNAQSRSTRARAVSAPVGDAPATSR	Tat-dependent (0.983 reliability score)
Unidentified protein (*Gluconobacter* YVTN beta-propeller repeat protein) (Ybp)	**MKFSHHAFIVSLVGLSLATAQATRA**QETPAAPAQAASAPATASSAASGVTSQAPASAATTPSAAPSSSATPTSTTAGSTTPASTAPVAPVVQVTPPAATSAATPVAGAVQTIPGMPAVIDPKNIYSETISGKISPAIKDDLARVYVPNLRG	Sec-dependent (0.988 reliability score)

The amino acid sequences listed here were fused directly to PhoA in the genomic library clones. Signal sequences within each fusion were identified using the PRED-TAT software [36]. Reliability scores were generated using combined scores from multiple prediction algorithms, with scores closer to 1 being the most reliable [37]. Signal sequences predicted by PRED-TAT are in bold. Protein identification was made using NCBI BLAST focusing on the *Asaia* SF2.1 genome sequence [38]. If a hypothetical protein was detected with the *Asaia* genome, another BLAST was performed using the entire NCBI database.

### Activity of cloned genes in *Asaia*

The unique clones from this screen were transformed into electrocompetent *Asaia* cells and plated on mannitol agar with BCIP and sodium phosphate. Eight of the clones (Tsr, Tdr, Gdh, Ybp, Csy, Amu, Ptp, and Aap) grew when transformed into *Asaia*, while the remaining eight did not grow after repeated attempts at transformation, indicating that they might be toxic when overexpressed in *Asaia*. The successfully transformed clones grew at varying rates and showed different levels of phosphatase activity on the BCIP agar plates when scored by eye.

**Fig 2:**
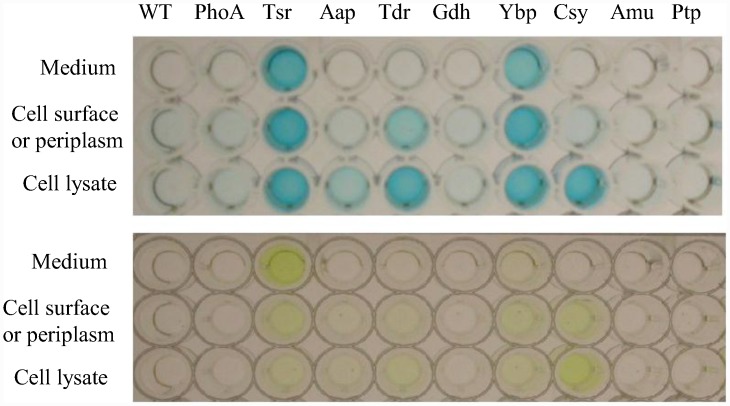
PhoA-*Asaia* protein fusion plasmids expressed in *Asaia*. PhoA = alkaline phosphatase with no signal sequence. The other lanes have *Asaia* sequences fused to ‘*phoA*. Tsr = TonB-dependent siderophore receptor. Aap = amino acid permease. Tdr = TonB-dependent receptor plug. Gdh = glucose dehydrogenase. Ybp = YVTN beta-propeller repeat protein. Csy = cellulose synthase. Amu = aminomutase. Ptp = peptide transport permease. The top ELISA used an anti-PhoA-HRP antibody to detect the presence of the alkaline phosphatase protein. The bottom plate assay used a PNPP substrate, which turns yellow when cleaved by active PhoA.

**Fig 3:**
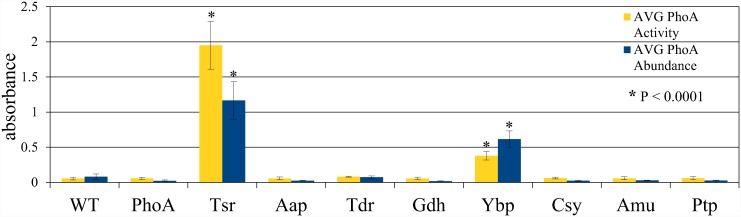
Secreted protein relative abundance and activity from PhoA-*Asaia* protein fusion plasmids expressed in *Asaia*. PhoA = alkaline phosphatase. Tsr = TonB-dependent siderophore receptor. Aap = amino acid permease. Tdr = TonB-dependent receptor plug. Gdh = glucose dehydrogenase. Ybp = YVTN beta-propeller repeat protein. Csy = cellulose synthase. Amu = aminomutase. Ptp = peptide transport permease. Both the ELISA and PNPP plates were repeated five times, and quantified using a plate reader. Signal intensity was read at 450 nm for the ELISA and at 400 nm for the PNPP assay. Although there was a similar amount of protein secreted by Tsr compared to Ybp, PhoA activity was more than five times as strong when secreted by Tsr. Each plate assay was performed 5 times and the means compared by an unpaired t-test using GraphPad Prism software, version 5.0.

Since BCIP penetrates the outer membrane of Gram-negative cells, PhoA could be mediating the color change as a secreted protein, as a membrane bound protein, or from within the periplasm [39]. To determine which, if any, of the candidates was actually secreted, an ELISA assay was performed using cleared growth medium, whole cells, or cell lysates. Two clones mediated efficient secretion into the supernatant. These were the TonB-dependent siderophore receptor protein (Tsr) and the YVTN beta-propeller protein (Ybp) (Fig 2, top). Another plate assay was prepared with PNPP substrate added, which turns yellow due to alkaline phosphatase activity (Fig 2, bottom). The relative amount of protein and activity in these plates were recorded using a plate reader (Fig 3 and S2 Table). The siderophore receptor appeared to secrete more PhoA and had a greater PhoA activity in the supernatant.

The siderophore receptor protein is an outer membrane pore complex that receives siderophores transporting metal ions [40]. The YVTN beta-propeller protein may be a structural protein that forms part of a protective S-layer on the outside of the cell [41]. Each of these proteins is typically localized at the cell surface in their native forms, but here they mediated secretion.

### Secretion of antiplasmodial effector proteins using *Asaia* signal sequences

**Fig 4:**
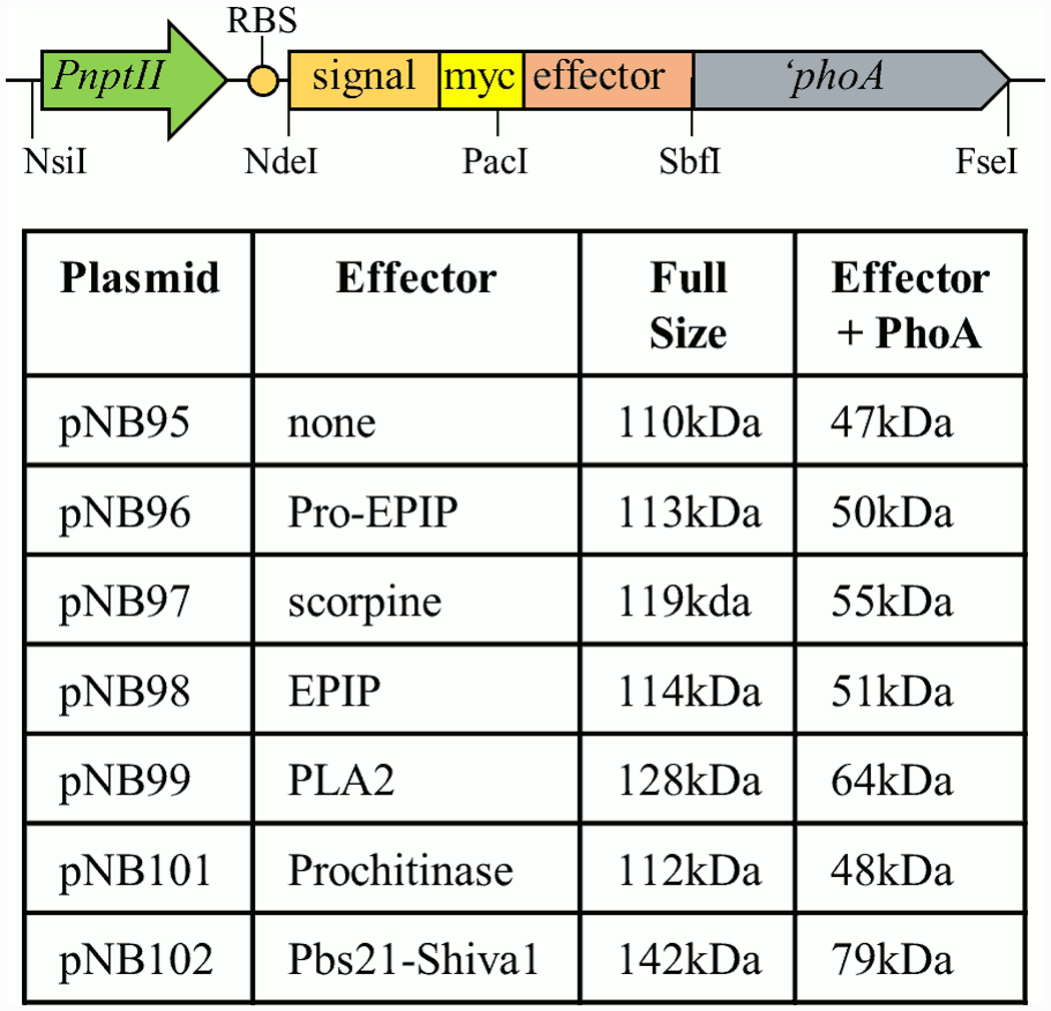
*Asaia* antiplasmodial expression plasmid. P*nptII* = constitutive promoter from *nptII*; RBS = ribosome binding site; signal = siderophore receptor or YVTN sequence from genomic clones; effector = antiplasmodial effector gene; ‘*phoA* = *phoA* without the native signal sequence.

The siderophore receptor and YVTN beta-propeller proteins were amplified from the library clones by PCR and cloned into pNB92, creating gene constructs that contain the gene for the secretion protein, a MYC epitope tag, a cloning site, and ‘*phoA* (Fig 4). Multiple antiplasmodial effector genes were cloned in-frame with the siderophore receptor gene and sequenced to confirm that there were no mutations. Manipulation of the YVTN beta-propeller protein plasmid proved difficult. Cloning attempts produced either no colonies on the selective plates or only false positives so this secretion signal was not pursued further.

**Fig 5:**
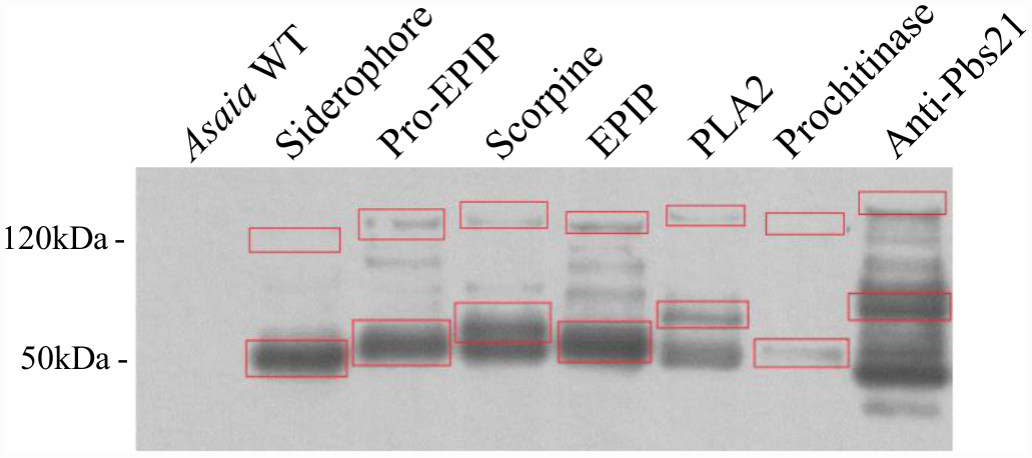
Western blot of *Asaia* culture supernatants detecting proteins secreted using the siderophore receptor fusion protein. Proteins were detected using a rabbit polyclonal anti-PhoA primary antibody and goat anti-rabbit HRP secondary. In each lane, red boxes highlight the predicted protein size of the full fusion proteins (higher molecular weight) and the predicted size of the effector molecules plus PhoA lacking the siderophore receptor protein (lower molecular weight).

*Asaia* SF2.1 cells were transformed with each of the siderophore receptor effector plasmids, and these strains were grown in mannitol broth supplemented with kanamycin to allow expression of the transgenes. Secretion of the fusion proteins was determined by western blot utilizing an antibody against the PhoA protein (Fig 5). Multiple products were detected in the supernatants of all constructs. Generally, the most abundant product was a small product corresponding in size to PhoA plus the effector protein. In each supernatant, higher molecular weight products could also be detected including some that migrated at the size of the siderophore receptor protein plus the effector and PhoA. The simplest explanation for these patterns is that the full-length protein is cleaved in multiple places within the siderophore receptor sequence, either in the periplasm or when the protein exits the cell, but that the PhoA portion is fairly stable and resistant to proteolysis. This makes sense considering PhoA is normally folded in the periplasm and then secreted.

### Activity of paratransgenic *Asaia* against *Plasmodium berghei*

**Fig 6:**
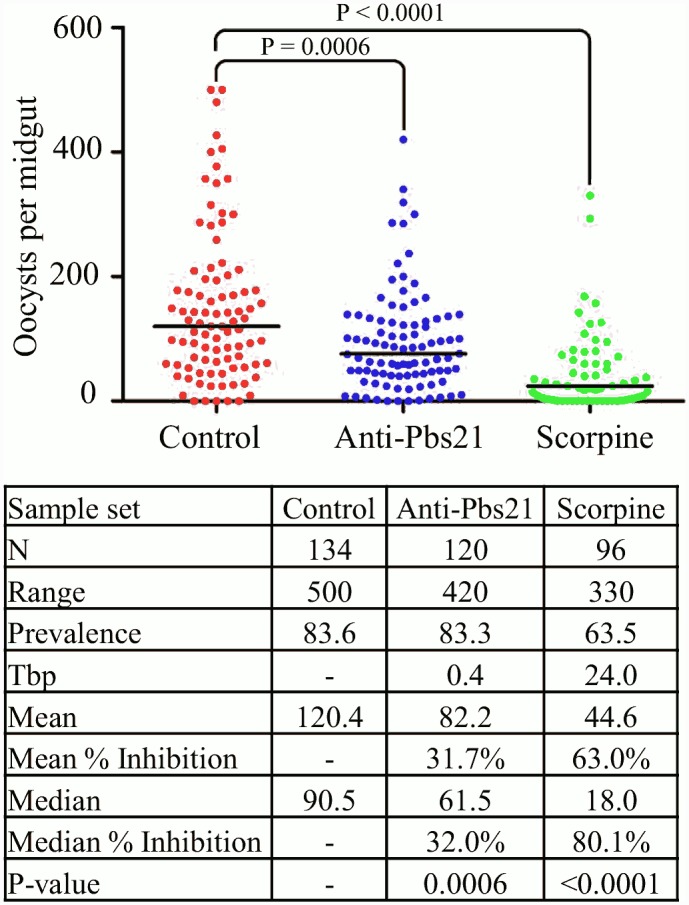
Disruption of *P*. *berghei* development by anti-Pbs21 scFv-Shiva1 immunotoxin and scorpine constructs. Three-day old *A*. *stephensi* mosquitoes were fed paratransgenic strains of *Asaia* expressing either a fusion constuct combining the siderophore receptor protein and PhoA (= control), or a fusion construct combining the siderophore receptor protein, an effector protein, and PhoA. These mosquitoes were then fed on an infective mouse and the parasite was allowed to develop for 14 days. The mosquitoes were then dissected, and oocysts on the midgut were counted for each individual. Each dot on the chart represents a single midgut count. The median number of oocysts for each data set is marked with a horizontal line. Inhibition = inhibition of oocyst formation relative to the control; Mean = mean oocyst number per midgut; Median = median oocyst number per midgut; N = number of mosquitoes analyzed; Prevalence = percentage of mosquitoes carrying at least one oocyst; Range = range of oocyst numbers per midgut; Tbp = transmission-blocking potential: 100 − {(prevalence of mosquitoes fed with recombinant *P*. *agglomerans*)/[prevalence of control mosquitoes] × 100}. The scorpine construct produced a significant inhibition of 80.1% calculated by the median oocyst number.

We tested two strains of *Asaia* that secreted effector proteins for their ability to inhibit the development of *P*. *berghei* in the midguts of female *A*. *stephensi* mosquitoes. These were the strain secreting scorpine and the strain secreting the anti-Pbs21 scFv-Shiva1 toxin fusion protein. These strains span a size spectrum from a small antiplasmodial peptide to a large complicated multi-domain protein. The paratransgenic strains were fed to *A*. *stephensi* mosquitoes, which were then challenged with an infective blood meal from a *P*. *berghei-*infected mouse two days later. Two weeks after the blood meal, the mosquitoes were dissected and oocysts per midgut were counted. The oocyst counts indicate a significant level of parasite killing from the scorpine effector, and a weaker effect from the anti-Pbs21 scFv-Shiva1 effector (Fig 6 and S3 Table). Both the anti-Pbs21 scFv-Shiva1 and the scorpine secretion strains significantly reduced the number of oocysts compared to control strains (unpaired t-test, p-value 0.0006 and <0.0001 respectively).

## Discussion

Malaria has plagued humans throughout history and is still a major cause of human morbidity and mortality [1]. Despite decades of research into interventions against the disease, it is still a widespread global problem. New technologies are needed to help reduce the burden that malaria causes and to hopefully eradicate the *Plasmodium* parasite entirely. Bacterial paratransgenesis is a relatively recent technology that has been proposed to combat human diseases, including Chagas disease, African sleeping sickness, HIV, and malaria [20, 42, 43, 15].

*Asaia* was proposed as a novel candidate for antiplasmodial paratransgenesis due to its favorable microbial ecology that allows it to colonize anopheline mosquito tissues and to transmit itself through the mosquito population [26]. The primary goal of this work was to modify *Asaia* to enable it to secrete proteins into the mosquito midgut and to hinder the life stages of *Plasmodium* that develop there following an infective blood meal. Interfering with this vulnerable stage of the malaria parasite may prevent human infection by rendering the mosquito an inefficient vector. The results here demonstrate the use of the *Asaia* sp. SF2.1 midgut symbiont of anopheline vector mosquitoes as a delivery vehicle for heterologous effector proteins.

The results of the alkaline phosphatase fusion library screen uncovered sixteen proteins that potentially mediated secretion from *Asaia* (Table 2). All of the proteins were homologous to either secreted or membrane-bound proteins in related bacterial species. These are all logical proteins to expect from a ‘*phoA* library screen since periplasmic, membrane-bound, surface-displayed, and secreted fusion proteins can all translocate the PhoA enzyme past the inner membrane where it can cleave the BCIP molecule used for screening [44]. These clones were further analyzed to determine exactly where each protein was localized.

The ELISA test for protein localization of PhoA fusion constructs demonstrated a high level of secretion mediated by the TonB-dependent siderophore receptor fusion protein and the YVTN repeat beta-propeller fusion protein, both of which are homologs of known bacterial membrane proteins [40, 41]. The alkaline phosphatase enzyme that was secreted by these systems was found to be active by a plate assay using PNPP. Since active alkaline phosphatase protein requires disulphide bond formation to achieve an active conformation [45], this means that the secreted proteins must spend time in the oxidizing environment of the periplasm before leaving the cell. PhoA secreted by the siderophore receptor fusion protein showed more robust phosphatase activity which indicates that the protein may have spent a greater amount of time in the periplasm and been allowed to achieve the correct conformation and form disulphide bonds. Disulphide bonds are important for the stability and activity of some single-chain antibody effectors that could be secreted by paratransgenic strains [46].

Western results (Fig 5) show that PhoA and the effector proteins fused to it secreted by the siderophore receptor pathway remain intact, while the *Asaia* siderophore receptor fusion protein is most likely degraded by periplasmic proteases [47]. This is expected, since the fusion protein only retains a portion of the original *Asaia* sequence, which likely cannot achieve its stable native conformation or insert into the membrane.

The in vivo inhibition tests demonstrate that the *Asaia* siderophore receptor protein is capable of mediating secretion of heterologous proteins efficiently enough to supply antiplasmodial effector proteins directly into the mosquito midgut environment despite the large size of the fusion proteins. These results compare favorably with previous paratransgenic efforts using *P*. *agglomerans* as the bacterial platform. When secreting scorpine + EPIP4, the *P*. *agglomerans* system reduced the prevalence of *P*. *berghei* in *A*. *stephensi* females by 11.1% and the median number of oocysts per midgut by 86.1% [15]. Expressing only scorpine, the *Asaia* strain reported here reduced prevalence by 20.1% and reduced the median number of oocysts per gut by 80.1%. Prevalence is the key measurement for successful paratransgenesis against malaria since a single oocyst can render a mosquito infective. By this measure the *Asaia* scorpine-secreting strain was approximately twice as effective as the *P*. *agglomerans* strain. Importantly, the high number of oocysts found in *P*. *berghei-*infected mosquitoes does not correlate with the level of infection that would be seen in other *Plasmodium* species in the field [10]. In the wild, individual *P*. *falciparum*-infected mosquitoes do not achieve infections of this magnitude, so the results of these tests are promising.

This research moves malaria paratransgenesis one step closer to practical use in the field. *Asaia* is a more useful symbiont of anopheline mosquitoes than previously considered paratransgenic species due to its beneficial microbial ecology. The antiplasmodial constructs tested in these experiments prove that *Asaia* can be effectively modified and used to deliver heterologous proteins into the mosquito midgut and to inhibit the sexual life stages of *P*. *berghei*.

## Supporting Information

S1 Table Oligonucleotides used in this study. (DOCX)

S2 Table Quantitative data from ELISA and PNPP assays for *Asaia*-PhoA fusion constructs. Both the ELISA and PNPP plates were repeated five times and quantified using a plate reader. Signal intensity was read at 450 nm for the ELISA and at 400 nm for the PNPP assay. Although there was a similar amount of protein secreted by Tsr compared to Ybp, PhoA activity was more than five times stronger when secreted by Tsr. PhoA = alkaline phosphatase. Tsr = TonB-dependent siderophore receptor. Aap = amino acid permease. Tdr = TonB-dependent receptor plug. Gdh = glucose dehydrogenase. Ybp = YVTN beta-propeller repeat protein. Csy = cellulose synthase. Amu = aminomutase. Ptp = peptide transport permease.
(DOCX)

S3 Table Oocyst counts from repeated antiplasmodial in vivo testing. (DOCX)
