# Supplementary material for: Inhibition of Plasmodium berghei Development in Mosquitoes by Effector Proteins Secreted from Asaia sp. Bacteria Using a Novel Native Secretion Signal
Source: PLoS One. 2015 Dec 4;10(12):e0143541. doi: 10.1371/journal.pone.0143541 (PMC4670117; doi:10.1371/journal.pone.0143541)
Supplement: S1 Table — (DOCX) [file pone.0143541.s001.docx]

**Table S1 Oligonucleotides used in this study**

| **Oligo** | **Sequence 5' - 3'** | **Purpose** |
| --- | --- | --- |
| pBBR rec F | ggaattgtgagcggataacaatttcacacaggaaacagctTTAACTTTAAGAAGGAGactgCATATGagtattcaacatttccg | Plasmid modification by bacterial recombination |
| pBBR rec R | taacaaaaatttaacgcgaattttaacaaaatattaacgcGAATTCggatccGGCCGGCCcctgcaggTTAATTAAggtctgacagttaccaatgc | '' |
| PnptII F NsiI | TAatgcatAACCGGAATTGCCAGCTGGG | Directional cloning of the promoter from *E. coli* neomycin phosphotransferase |
| PnptII R NdeI | taCATATGTTTTTCCTCCTTATAAAGTTAATC | '' |
| PhoA F NdeI | taCATATGcctgttctggaaaaccgggc | Directional cloning of *E. coli* alkaline phosphatase |
| PhoA R PacI | taTTAATTAAggtgacaaggcaggaaaccac. | '' |
| PhoA F NdePacSbf | TACATATGTTAATTAAAACCTGCAGGATGCCTGTTCTGGAAAACCGGGCTG | phoA directional cloning to generate effector expression constructs |
| PhoA R Fse | taGGCCGGCCggtgacaaggcaggaaaccac | '' |
| siderophore F NdeI | TAcatATGCCCAGAGCCGCTCGAC | secretory leader protein directional cloning |
| siderophore R MYC PacI | TAttaattaaGTTCAGATCTTCCTCCGAGATCAGTTTCTGTTCAACAATCGGCGCCTCAGCCTGG | '' |
| YVTN F NdeI | TAcatATGAAGTTTTCACATCACGC | '' |
| YVTN R MYC PacI | TAttaattaaGTTCAGATCTTCCTCCGAGATCAGTTTCTGTTCAATGGCATAGGCCACATCATC | '' |
| scorpine F PacI | taTTAATTAAaggctggattaacgaagagaagattc | effector gene  directional cloning |
| scorpine R SbfI | taCCTGCAGGaataagacaggggggtgccgc | '' |
| Shiva1 F | taTTAATTAAaatgccgcgttggcgtctgttc | '' |
| Shiva1 R | taCCTGCAGGaacccaccgcacgcgcatc | '' |
| PRO-EPIP F PacI | taTTAATTAAaatggctagcgaagaaccgc | '' |
| PRO-EPIP R SbfI | taCCTGCAGGacccggggctgcctttcac | '' |
| Pbs21Shiva F PacI | taTTAATTAAaagcgaggtccagctgcagcagag | '' |
| Pbs21Shiva R SbfI | taCCTGCAGGaacccaccgcacgcgcatc | '' |
| mPLA2 F PacI | taTTAATTAAaatggctagctggcagatcc | '' |
| mPLA2 R SbfI | taCCTGCAGGagtacttgcgcagatcgaacc | '' |
| EPIP4 F PacI | taTTAATTAAagataaatccctggttaagg | '' |
| EPIP4 R SbfI | taCCTGCAGGagcccggcgagcccttc | '' |
| Prochitinase F PacI | taTTAATTAAagaagaaccgcataaggccg | '' |
| Prochitinase R SbfI | taCCTGCAGGacttcttgccttcggcgctg | '' |
