# Supplementary material for: Inhibition of Plasmodium berghei Development in Mosquitoes by Effector Proteins Secreted from Asaia sp. Bacteria Using a Novel Native Secretion Signal
Source: PLoS One. 2015 Dec 4;10(12):e0143541. doi: 10.1371/journal.pone.0143541 (PMC4670117; doi:10.1371/journal.pone.0143541)
Supplement: S2 Table — Both the ELISA and PNPP plates were repeated five times and quantified using a plate reader. Signal intensity was read at 450 nm for the ELISA and at 400 nm for the PNPP assay. Although there was a similar amount of protein secreted by Tsr compared to Ybp, PhoA activity was more than five times stronger when secreted by Tsr. PhoA = alkaline phosphatase. Tsr = TonB-dependent siderophore receptor. Aap = amino acid permease. Tdr = TonB-dependent receptor plug. Gdh = glucose dehydrogenase. Ybp = YVTN beta-propeller repeat protein. Csy = cellulose synthase. Amu = aminomutase. Ptp = peptide transport permease. (DOCX) [file pone.0143541.s002.docx]

**Table S2. Quantitative data from ELISA and PNPP assays for *Asaia*-PhoA fusion constructs**

|  | ***Asaia* WT** | **PhoA** | **Tsr** | **Asp** | **Tdr** | **Gdh** | **Ybp** | **Csy** | **Amu** | **Ptp** |
| --- | --- | --- | --- | --- | --- | --- | --- | --- | --- | --- |
| **AVG PhoA abundance** | 0.081 (+/-0.038) | 0.023 (+/- 0.012) | 1.167 (+/- 0.266) | 0.026 (+/- 0.004) | 0.075 (+/- 0.016) | 0.019 (+/- 0.003) | 0.616 (+/- 0.117) | 0.025 (+/- 0.008) | 0.029 (+/- 0.004) | 0.028 (+/- 0.005) |
| **AVG PhoA activity** | 0.055 (+/- 0.020) | 0.058 (+/- 0.014) | 1.951 (+/- 0.342) | 0.057 (+/- 0.020) | 0.081 (+/- 0.007) | 0.057 (+/- 0.017) | 0.380 (+/- 0.062) | 0.062 (+/- 0.012) | 0.059 (+/- 0.021) | 0.060 (+/- 0.023) |

Both the ELISA and PNPP plates were repeated five times and quantified using a plate reader. Signal intensity was read at 450 nm for the ELISA and at 400 nm for the PNPP assay. Although there was a similar amount of protein secreted by Tsr compared to Ybp, PhoA activity was more than five times stronger when secreted by Tsr. PhoA = alkaline phosphatase. Tsr = TonB-dependent siderophore receptor. Aap = amino acid permease. Tdr = TonB-dependent receptor plug. Gdh = glucose dehydrogenase. Ybp = YVTN beta-propeller repeat protein. Csy = cellulose synthase. Amu = aminomutase. Ptp = peptide transport permease.
