# Supplementary material for: Inhibition of Plasmodium berghei Development in Mosquitoes by Effector Proteins Secreted from Asaia sp. Bacteria Using a Novel Native Secretion Signal
Source: PLoS One. 2015 Dec 4;10(12):e0143541. doi: 10.1371/journal.pone.0143541 (PMC4670117; doi:10.1371/journal.pone.0143541)
Supplement: S3 Table — (DOCX) [file pone.0143541.s003.docx]

**Table S3. Oocyst counts from repeated anti-*Plasmodium* in vivo testing.**

| **Control** | | **Control** | | **anti-Pbs21-Shiva1** | | **anti-Pbs21-Shiva1** | | **Scorpine** | |  | **Scorpine** | |
| --- | --- | --- | --- | --- | --- | --- | --- | --- | --- | --- | --- | --- |
| 24 | 175 | 44 | 209 | 60 | 0 | 420 | 122 | 27 | 2 | 21 | | 82 |
| 26 | 143 | 259 | 500 | 44 | 151 | 10 | 87 | 30 | 38 | 3 | | 0 |
| 28 | 40 | 66 | 148 | 41 | 28 | 98 | 31 | 0 | 0 | 95 | | 24 |
| 28 | 38 | 115 | 169 | 0 | 4 | 195 | 49 | 60 | 51 | 35 | | 24 |
| 120 | 194 | 93 | 175 | 59 | 31 | 0 | 119 | 168 | 28 | 15 | | 60 |
| 93 | 282 | 142 | 107 | 45 | 49 | 90 | 8 | 0 | 8 | 126 | | 2 |
| 287 | 222 | 211 | 0 | 49 | 58 | 107 | 6 | 293 | 0 | 142 | | 1 |
| 157 | 160 | 427 | 0 | 41 | 100 | 300 | 5 | 0 | 21 | 330 | | 45 |
| 101 | 97 | 53 | 53 | 62 | 87 | 67 | 285 | 108 | 4 | 0 | | 6 |
| 24 | 9 | 9 | 111 | 86 | 40 | 237 | 63 | 0 | 18 | 8 | | 0 |
| 36 | 120 | 196 | 377 | 26 | 49 | 110 | 49 | 18 | 5 | 98 | | 0 |
| 68 | 70 | 149 | 500 | 47 | 20 | 84 | 43 | 14 |  | 101 | | 180 |
| 61 | 125 | 86 | 88 | 101 | 44 | 126 | 104 | 71 |  | 132 | | 73 |
| 45 | 59 | 142 | 0 | 76 |  | 8 | 122 | 80 |  | 111 | | 116 |
| 202 | 98 | 357 | 86 | 93 |  | 286 | 340 | 157 |  | 151 | | 76 |
| 58 |  | 302 | 111 | 23 |  | 221 | 69 | 124 |  | 64 | | 18 |
| 130 |  | 315 | 133 | 69 |  | 7 | 2 | 40 |  | 120 | | 28 |
| 82 |  | 405 | 350 | 96 |  | 200 | 177 | 20 |  | 106 | | 30 |
| 44 |  | 60 | 480 | 99 |  | 166 | 1 | 66 |  | 30 | | 114 |
| 178 |  | 287 | 400 | 97 |  | 93 | 154 | 79 |  | 64 | | 77 |
| 95 |  | 72 | 140 | 61 |  | 139 | 159 | 74 |  | 180 | | 4 |
| 54 |  | 170 | 214 | 19 |  | 136 | 138 | 33 |  | 9 | | 35 |
| 63 |  | 128 | 0 | 132 |  | 57 | 103 | 0 |  | 65 | |  |
| 167 |  | 357 | 300 | 129 |  | 75 | 24 | 0 |  |  | |  |
| 178 |  | 144 |  | 133 |  | 139 | 189 | 60 |  |  | |  |
| 0 |  |  |  | 61 |  | 52 | 319 | 0 |  |  | |  |
| 144 |  |  |  | 128 |  | 166 |  | 41 |  |  | |  |
|  |  |  |  |  |  |  |  |  |  |  | |  |
